# Clinal Variation in Short Tandem Repeats Linked to Gene Expression in Sunflower (*Helianthus annuus* L.)

**DOI:** 10.3390/biom14080944

**Published:** 2024-08-03

**Authors:** Chathurani Ranathunge, Mark E. Welch

**Affiliations:** Department of Biological Sciences, Mississippi State University, Starkville, MS 39762, USA; mw497@msstate.edu

**Keywords:** microsatellite, short tandem repeats, STR, sunflower, *Helianthus annuus*, RNA-Seq, gene expression, adaptation, CHUP1

## Abstract

Short tandem repeat (STR) variation is rarely explored as a contributor to adaptive evolution. An intriguing mechanism involving STRs suggests that STRs function as “tuning knobs” of adaptation whereby stepwise changes in STR allele length have stepwise effects on phenotypes. Previously, we tested the predictions of the “tuning knob” model at the gene expression level by conducting an RNA-Seq experiment on natural populations of common sunflower (*Helianthus annuus* L.) transecting a well-defined cline from Kansas to Oklahoma. We identified 479 STRs with significant allele length effects on gene expression (eSTRs). In this study, we expanded the range to populations further north and south of the focal populations and used a targeted approach to study the relationship between STR allele length and gene expression in five selected eSTRs. Seeds from 96 individuals from six natural populations of sunflower from Nebraska and Texas were grown in a common garden. The individuals were genotyped at the five eSTRs, and gene expression was quantified with qRT-PCR. Linear regression models identified that eSTR length in comp26672 was significantly correlated with gene expression. Further, the length of comp26672 eSTR was significantly correlated with latitude across the range from Nebraska to Texas. The eSTR locus comp26672 was located in the CHUP1 gene, a gene associated with chloroplast movement in response to light intensity, which suggests a potential adaptive role for the eSTR locus. Collectively, our results from this targeted study show a consistent relationship between allele length and gene expression in some eSTRs across a broad geographical range in sunflower and suggest that some eSTRs may contribute to adaptive traits in common sunflower.

## 1. Introduction

Significant portions of eukaryotic genomes are composed of repetitive DNA sequences. Research now implicates repetitive sequences in numerous functional processes [1,2]. More recently, it has been suggested that repeats may hold the key to understanding the unexplained heritability of traits in most models involving SNPs [3]. Hyper-variable microsatellites or short tandem repeats (STRs) are particularly interesting in this regard. An intriguing model involving STRs suggests that they may function as evolutionary “tuning knobs” facilitating organismal evolvability by altering phenotypes in a stepwise manner [4,5,6]. STRs possess a repertoire of favorable features as potential drivers of rapid adaptive evolution [7]; STRs have mutation rates that are orders of magnitude greater than base substitution rates [8], and they are found abundantly within functional regions of genomes [9,10].

Recent large-scale genomic and transcriptomic studies have shed light on the extent to which functional STRs may exist in organismal genomes and their potential roles [3,11,12,13,14,15]. Studies suggesting a potential adaptive role for STRs have linked STR variation to reproductive success in some mammals [16], neuronal and craniofocal development in primates [17], flowering time variation in plants [18,19,20], and immune response in plants [12], among others. Further, some studies have revealed that variation in STR tract lengths could be linked to variation in environmental gradients [13]. To this end, clinal patterns of variation in STR tract lengths have been previously reported in several organisms, with the great majority of evidence coming from STRs associated with the circadian rhythm of organisms across latitudinal gradients [21,22,23,24,25]. On the whole, despite these sporadic studies, the extent to which STRs, especially those that that are linked to adaptive traits, could be shaped by the environment remains largely unexplored.

**Figure 1:**
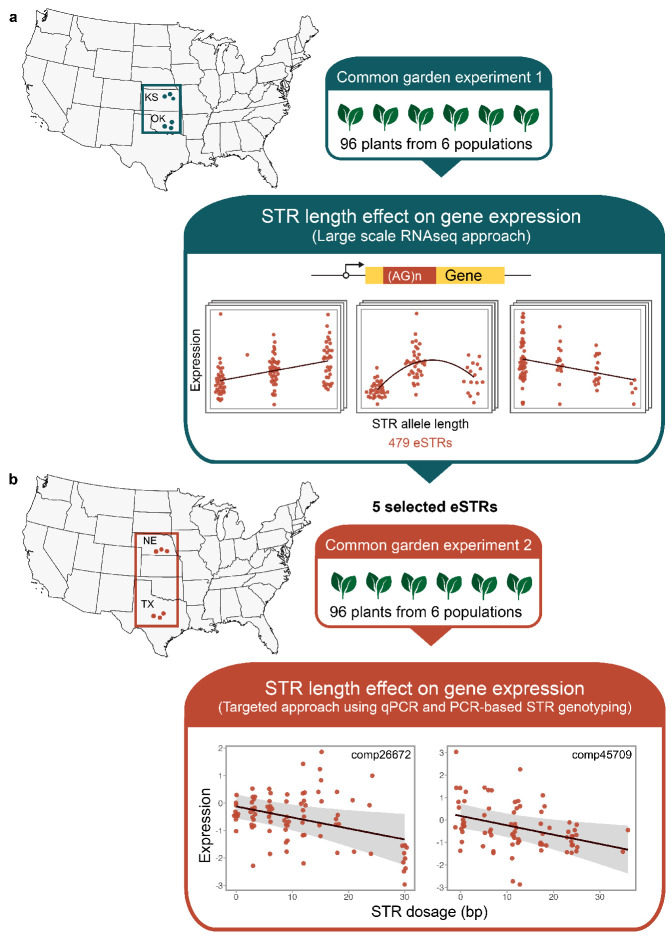
Functional STRs in sunflower. (**a**) Previously, an RNA-Seq experiment conducted on 95 plants from a narrow latitudinal range from Kansas (KS) to Oklahoma (OK) grown in a common garden identified 479 STRs with significant allele length effects (ANCOVA, adj. *p*−value < 0.05) on gene expression (termed eSTRs) [14]. (**b**) A targeted approach was used to study five of the previously identified eSTRs across populations of sunflower from further north (Nebraska−NE) and south (Texas−TX). STRs were genotyped with PCR techniques, and gene expression at the eSTR-containing genes were quantified with qPCR. Two loci−comp26672 located in the CHUP1 gene and comp45709 located in the CYP86A22 gene−showed significant effects of STR length on gene expression.

Previously, using an RNA-Seq approach, we identified 479 STRs linked to gene expression (hereafter referred to as significant expression STRs or eSTRs) in natural sunflower (*Helianthus annuus* L.) populations across a well-defined cline from Kansas to Oklahoma [14] (Figure 1a). A population genetic study conducted on sunflower populations along the same cline revealed that some eSTRs may be under directional selection [26]. In the current study, we use a targeted approach to study five selected eSTRs (of the 479) located in potential candidate genes for adaptation in sunflowers. We explore the relationship between eSTR length and gene expression in sunflower populations across a broader latitudinal range with populations further north and south of the focal population used in [14] (Figure 1b). Further, we explore the extent to which eSTR variation is shaped by the environment by assessing the correlation between eSTR length and latitude. Our results show that an eSTR located in a gene involved in chloroplast movement (CHUP1) is significantly associated with gene expression. In the CHUP1 gene, we show that shorter eSTR lengths, particularly favored in southern latitudes, are linked to higher expression levels. Finally, we explore these results in the context of the adaptive landscape of sunflower evolution across this well-defined cline [27,28,29].

## 2. Materials and Methods

### 2.1. Sample Collection and Common Garden Experiment

Seeds from three populations of *H. annuus* collected from the wild in Nebraska were obtained from the USDA’s North Central Regional Plant Introduction Station (Ames, IA, USA). Seeds from three wild *H. annuus* populations in Texas were collected, and the vouchers were deposited at the Mississippi State University Herbarium (Supplemental Table S1). Seeds were scarified and germinated on moist filter paper in Petri dishes. Seeds were grown in 2.54 cm “cone-tainers” (Stuwe & Sons, Inc., Tangent, OR, USA). The “cone-tainers” were arranged in a randomized block design and kept in a greenhouse under controlled conditions for five weeks.

### 2.2. RNA Extraction

Young leaves from five-week-old plants were collected for RNA extraction. A total of 96 individuals representing the six populations were used in the study. Sixteen individuals (biological replicates) per population were used. RNA was isolated from 20 mg of fresh leaf tissue with Maxwell 16 LEV simplyRNA Tissue kits (Promega, WI, USA). Isolated RNA samples were converted to cDNA using a High-Capacity cDNA Reverse Transcription kit with RNase inhibitor (Applied Biosystems, Foster City, CA, USA).

### 2.3. Gene Expression Quantification

Previously, we identified 479 eSTRs with a significant allele length effect on gene expression across populations of sunflowers from two latitudinal locations in Kansas and Oklahoma [14]. Five of the previously identified eSTRs were selected to assess the effect of eSTR length on gene expression in populations further north (Nebraska) and south (Texas) of the previously sampled locations. The putative functions of the selected eSTR-containing genes from the BLASTX search against the *Helianthus annuus* protein sequence database [14] are given in Table 1. The five eSTRs for this study were selected on the basis of the magnitude of the effect size of eSTR length on gene expression variation, the presence of fewer alleles at a locus that facilitates accurate genotyping [26], and the potential role of the eSTR-associated genes in plant adaptation (Table 1).

Two constitutively expressed genes, actin and ubiquitin, were selected as standards for estimating the relative concentrations of the five eSTR-containing genes. TaqMan assays for the two standards were previously designed [30], and new assays were synthesized for the five selected eSTR-containing genes with Primer Express v.3.0 (Applied Biosystems, Foster City, CA, USA) (Supplemental Table S2). The assay probes were ZEN double-quenched probes that contained an internal quencher, a 3′ Iowa Black forward quencher (IABkFQ), and a 5′ 6-FAM reporter (Integrated DNA Technologies, Coralville, IA, USA). Standard curves were generated for the seven assays with six-point, 1:1 serial dilutions of cDNA samples from four individuals representing four of the six populations. Real-time PCR (qPCR) was carried out on an ABI StepOne Real-time PCR System (Applied Biosystems). The reaction mix included 5 µL (1X concentration) of 2X iTaq supermix with ROX (Bio-Rad, Hercules, CA, USA), 1 µL (1X concentration) of the TaqMan assay, 3 µL of ddH_2_O, and 1 µL of the cDNA sample. The amplification profile consisted of a 2 min hold at 50 °C, an initial denaturation step at 95 °C for 3 min, 40 cycles of denaturation at 95 °C for 30 s, and an annealing and elongation step at 72 ° C for 40 s. The cycle threshold (C_T_) values obtained from the qPCR runs were used to generate standard curves for each of the seven assays (Supplemental Table S3). ANCOVAs were conducted with individuals as the discrete and the log_2_-transformed concentration as the continuous explanatory variables, respectively. Models for each of the seven assays were established to quantify relative concentrations from the C_T_ values (Supplemental Tables S4 and S5) for the 96 individuals. Actin and ubiquitin concentrations were averaged to calculate the standard concentration for each individual. Log_2_ transformed concentrations for each of the eSTR-containing gene assays were regressed against the standard concentration to calculate the standardized residual concentrations, which were then used in downstream analysis.

### 2.4. DNA Extraction and STR Genotyping

Approximately 15–20 mg of dried leaf tissue from the 96 plants used in the qPCR experiment were macerated using the Retsch MM200 ball mill (Retsch Incorporated, Newtown, PA, USA). DNA was extracted using the Maxwell 16 tissue DNA purification kit (Promega, Madison, WI, USA). Primers previously designed for the five eSTRs [26] were used to conduct three-primer PCR. Touchdown PCR [31] was performed as explained in [26]. Fragment analysis was performed on ABI 3730 capillary sequencers (Applied Biosystems) at the Arizona State University DNA laboratory using LIZ-500 as the size standard (GeneScan—500 LIZ Size Standard—Applied Biosystems). STR genotypes were scored using GeneMarker version 2.6.7 (SoftGenetics) (Supplemental Table S6).

### 2.5. Effect of eSTR Length on Gene Expression

Extracting STR repeat unit numbers directly from amplicon lengths could be erroneous [32]. Therefore, we calculated normalized STR allele lengths for each individual at an eSTR locus by first subtracting the amplicon length of the shortest allele observed for each locus from the amplicon length and then adding the two allele lengths together. A similar approach was used by [33], and the calculated combined allele length was referred to as “STR dosage”. The combined allele length at a STR locus or STR the dosage can be calculated with the following formula, where Y_ij_ represents combined allele length for ith individual at jth locus when X_1,ij_, X_2,ij_ and X_mj_ represent the amplicon lengths of the two alleles and the shortest allele length observed at the jth locus, respectively.

$$Y_{ij}=\left(X_{1,ij}-X_{mj}\right)+\left(X_{2,ij}-X_{mj}\right)$$

Previous analyses show that the five eSTRs used in this study tend to show a linear relationship between the STR allele length and the gene expression [14]. Therefore, we limited our analysis to investigating the possible linear relationship between the STR length and gene expression, although other empirical studies provide evidence for non-linear relationships as well [34]. We performed linear regression between log_2_-transformed gene expression and STR dosage with the population as a categorical covariate for each of the five eSTRs. Statistical analyses were performed in R statistical software (version 4.2.1) [35]. To estimate the relative contributions of STR dosage and population on gene expression, Type II ANOVA was performed with the “Anova” function in the R package car [36]. The regression models were visualized using the "visreg" function in the R package visreg [37].

### 2.6. Latitudinal Variation in eSTR Length

To cover the latitudinal range from Texas to Nebraska, in addition to the six populations from Texas and Nebraska used in this study, we included six more sunflower populations, three each from Kansas and Oklahoma, from [26]. First, we calculated normalized STR allele length for each of the five eSTR loci. Normalized STR length for the ith individual for the kth allele of the jth locus was calculated by subtracting the shortest allele length (Xm) observed at jth locus from each allele length (X).

$$Y_{ijk}=X_{ijk}-X_{mj}$$

We built linear regression models using the normalized eSTR allele length as the response variable, latitude, and population as predictor variables. The regression models were visualized using the “visreg” function in the R package visreg [37].

All statistical analyses were performed using the R Statistical software (version 4.2.1; [35]) using the packages visreg (version 2.7.0; [37]), car (version 3.1.0; [36]), reshape2 (version 1.4.4; [38]), and report (version 0.5.5; [39]).

## 3. Results

### 3.1. Gene Expression Variation in eSTR-Containing Genes

To estimate the correlation between the two constitutively expressed genes, actin and ubiquitin, used as the standards, we built linear regression models with log_2_-transformed concentrations of the two genes. The analyses revealed a strong linear relationship between the concentrations of the two standards, as expected (R^2^ = 0.91, *p* < 0.0001) (Supplemental Figure S1). This strong correlation detected between the two standards suggests that using either one of the two genes as the control gene for the normalizing expression is appropriate. However, we opted to use both to improve accuracy. When the concentrations of the five eSTR-containing genes were regressed against the average concentrations of the two standards, four of them each revealed a positive correlation between the concentrations with coefficients of the correlation (R^2^) ranging from 0.22 to 0.82 (Supplemental Table S7) (Supplemental Figure S2).

### 3.2. The Relationship between the eSTR Length and Gene Expression

**Figure 2:**
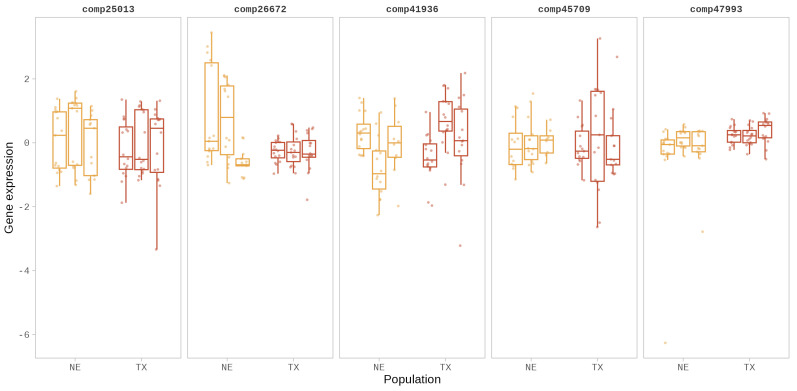
Gene expression variation in the five eSTR-containing genes across the sunflower populations from Nebraska (NE) and Texas (TX) used in the study (Supplemental Table S12). A total of 96 individuals representing the six populations (16 individuals per population) were used in the study.

We fitted linear regression models on the five eSTR-containing genes to estimate the proportion of gene expression variation explained by the STR length or dosage and population (formula: log-transformed gene expression ∼ STR dosage + population). Of the models built using the five eSTR-containing genes, one model (comp26672) explained a statistically significant (R^2^ = 0.33, *p* < 0.001, adj. R^2^ = 0.28) and substantial proportion of variance in gene expression. Models built for two loci, comp41936 (R^2^ = 0.24, *p* < 0.001, adj. R^2^ = 0.19) and comp47993 (R^2^ = 0.16, *p* = 0.021, adj. R^2^ = 0.10), explained a statistically significant and moderate proportion of variance in gene expression. The remaining two models (comp45709 and comp25013) were statistically non-significant (Supplemental Table S8). In the models built for eSTR loci comp26672 and comp45709, we observed statistically significant relationships between STR dosage and gene expression (Figure 1b), while models built for loci comp25013, comp41936, and comp47993 did not show a significant relationship between STR dosage and gene expression (Supplementary Table S8). Across the five eSTRs, the estimated effect size (partial eta^2^) of STR dosage on gene expression ranged between 0.003 and −0.09 (Supplemental Table S9). Our results show that the effect size (partial Eta^2^) of population on gene expression variation ranged from 0.037 to 0.31 across the five eSTR-containing genes (Supplemental Table S9) with a significant proportion of the variation in gene expression in the loci comp26672, comp41936, and comp47993 explained by population differences (Figure 2).

Within the model built for eSTR locus comp26672, the effect of STR dosage on gene expression was statistically significant and negative (β = −0.04, *p* = 0.013), and the effects of populations, NE6 (β = −1.51, *p* < 0.001), TX1 (β = −1.59, *p* < 0.001), TX2 (β = −1.52, *p* < 0.001), and TX3 (β = −1.52, *p* < 0.001) were statistically significant and negative (Supplemental Table S8). The model built for comp45709 was statistically not significant, and only a weak proportion of variance in gene expression was explained by the model (R^2^ = 0.10, *p* = 0.283, adj. R^2^ = 0.02). However, within this model, the effect of STR dosage on gene expression was statistically significant and negative (β = −0.04, *p* = 0.011) (Figure 1b) (Supplemental Table S8). Within the model built for the eSTR locus comp41936, the effects of population NE5 (β = −1.10, *p* = 0.002) and TX1 (β = −0.77, *p* = 0.022) were statistically significant and negative. In the model built for locus comp47993, populations NE5 (β = 0.66, *p* = 0.024), TX1 (β = 0.70, *p* = 0.014), TX2 (β = 1.01, *p* = 0.004), and TX3 (β = 0.97, *p* < 0.001) showed statistically significant, positive effects on gene expression (Supplemental Table S8).

### 3.3. Clinal Variation in eSTR Length

We fitted the linear regression models to predict normalized eSTR length with latitude (formula: normalized eSTR length ∼ latitude). The models built for loci comp26672 (R^2^ = 0.07, *p* < 0.001, adj. R^2^ = 0.07), comp25013 (R^2^ = 0.03, *p* < 0.001, adj. R^2^ = 0.02), and comp47993 (R^2^ = 0.02, *p* < 0.001, adj. R^2^ = 0.02) explained a statistically significant but weak proportion of variance in STR length (Supplemental Table S10). The model built for locus comp45709 (R^2^ = 0.01, *p* = 0.003, adj. R^2^ = 0.01) was statistically significant but only explained a very weak proportion of variance in STR length. The model built for comp41936 was statistically non-significant. In the models built for eSTR loci comp26672, comp25013, comp47993, and comp45709, we observed statistically significant effects of latitude on the normalized eSTR length, while in the models built for the locus comp41936, the relationship between latitude and eSTR length was statistically non-significant (Supplementary Table S10). In comp26672, the effect of latitude on normalized eSTR length was statistically significant and positive (β = 0.45, *p* < 0.001) (Supplemental Table S10). Similar significant positive trends were observed in the linear models built for comp25013 (β = 0.37, *p* < 0.001) and comp45709 (β = 0.22, *p* = 0.003), while in the model built for comp47993, the effect of latitude on eSTR length was statistically significant and negative (β = −0.27, *p* < 0.001) (Supplemental Table S10). Results from the ANOVA showed that the estimated effect size (partial Eta^2^) for latitude on eSTR length ranged between 0.00008 to 0.074 (Supplemental Table S11).

## 4. Discussion

STRs have long been considered neutral regions of the genome with no significant phenotypic consequences. On the contrary, research now provides a wealth of evidence to show that STRs can have significant effects on phenotypes of many organisms [12,14,33,40]. Previously, we identified 479 eSTRs in the common sunflower with significant effects of STR length on phenotype at the gene expression level [14]. Further, in a subset of these eSTRs we detected signatures of directional selection across a well-defined cline in sunflower, which suggested that some of these eSTRs could have a potentially adaptive role in sunflower evolution [26]. To understand this functional and potentially adaptive role of eSTRs, in the current study, we used a more targeted approach to explore the relationship between eSTR length and gene expression at five eSTR-containing genes across a broader latitudinal range to that of the focal study. Sampling these populations further north and south of the focal populations also allowed us the opportunity to test the hypothesis that shorter or longer allele lengths at eSTRs may be favored in populations in even more extreme conditions than that of the focal populations. We found that eSTR locus comp26672 in the CHUP1 gene associated with choloroplast movement has a significant linear relationship between STR length and gene expression. Further, the length of CHUP1-associated eSTR correlates significantly with latitude, which suggests that shorter or longer eSTR lengths may be favored in extreme conditions.

**Figure 3:**
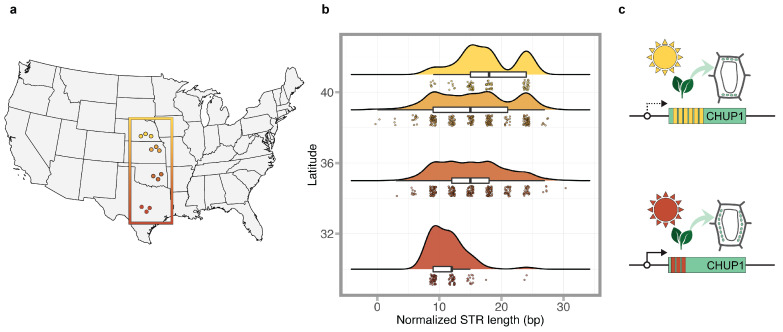
eSTR located in the Chloroplast Unusual Postioning 1 (CHUP1) gene. (**a**) Location map of the sunflower populations used to test the effect of latitude eSTR length. (**b**) The variation in length in the CHUP1-associated eSTR across the four latitudinal locations used in the study. (**c**) The activity of CHUP1 under different light intensities. Under high light conditions, chloroplasts move toward anticlinal walls to minimize potential photodamage. Shorter eSTR lengths associated with higher levels of CHUP1 expression appear to be favored in southern latitudes where photodamage due to long periods of high light intensity is likely to occur.

Our findings from the current study suggest that the eSTR located in the CHUP1 gene could be particularly useful in our quest to understand how STRs may be involved in the adaptive evolution of the common sunflower. The chloroplast’s unusual positioning 1 (CHUP1) gene was first identified by [41] as a unique gene that produces a protein essential for organellar positioning and movement within plant cells. The movement of chloroplasts in response to light is particularly interesting in this regard. Under low light conditions, chloroplasts are located along the periclinal cell walls, maximizing their potential to harvest sufficient sunlight. Under high light conditions, chloroplasts move toward anticlinal walls to minimize potential photodamage (Figure 3c). If chloroplasts are not redistributed normally under continuous high light intensity, it could result in severe photodamage and necrosis [42]. In line with this evidence, it is reasonable to assume that plants growing in environments where periods of high light intensity are long should evolve regulatory mechanisms to minimize photodamage. In such environments where efficient relocation of chloroplasts is essential for survival, higher levels of expression of genes such as CHUP1 may be favored. In the current study, we detected a significant STR length effect on the expression of the CHUP1 gene with shorter alleles associated with higher levels of gene expression (Figure 1b). Furthermore, in the eSTR located in the CHUP1 gene, we detected a significant positive correlation between the normalized eSTR length and latitude, with shorter alleles being favored in southern (lower) latitudes (Figure 3b). Together, our results from the CHUP1-associated eSTR suggest that shorter STR lengths associated with higher levels of expression in CHUP1 may be favored in southern latitudes, where photodamage due to long periods of high light intensity is likely to occur. Similarly, longer lengths of the CHUP1-associated eSTR linked to lower levels of CHUP1 expression may be favored in populations in the north, where periods of high light intensity are relatively shorter compared to southern latitudes (Figure 3). Interestingly, the eSTR identified within the CHUP1 gene is located in its coding region (Table 1). The location of eSTRs within genes is important in understanding the mechanisms by which eSTRs may regulate gene expression. Several studies have presented evidence of eSTRs located in coding regions [11,14,33]. While much is known about likely regulatory mechanisms involving eSTRs in the UTRs [43,44], cis-regulatory mechanisms involving eSTRs in coding regions are relatively unknown. One mechanism involving triplet repeats in general changes in links in the length of the repeat tracts to changes in nucleosome binding, which can affect gene expression [45,46]. Results from the current study and those from previous studies [11,14,33] warrant further explorations of the coding region eSTRs to understand their role in cis-regulation of gene expression.

Despite our results suggesting a significant correlation between STR length and gene expression in the CHUP1-associated eSTR (comp26672), results from the remaining four loci fail to sufficiently capture the association between STR length and gene expression we observed across a narrow latitudinal range in the previous large-scale transcriptomic study [14]. In spite of the popularity of transcriptomic approaches, they are often criticized for their limited ability to identify candidate loci for adaptation [47]. Critics of transcriptomics point out that large changes in gene expression may not necessarily cause large effects on fitness and that gene expression is often an unreliable indicator of protein activity [47]. In response to this criticism, it has been suggested that large-scale transcriptomic studies should be used as means to inform subsequent functional studies targeting specific loci to assess their importance in adaptation [48]. Certainly, the targeted approach we used in the current study appears to have helped screen results from the large scale transcriptomic study for eSTRs that may have a significant impact on sunflower adaptation. However, to capture the full scale of the contribution of STRs to sunflower adaptation, further experimental and functional studies targeting specific eSTRs may be needed.

## Figures and Tables

**Table 1:** eSTR motif, location of the eSTR within the gene, and the putative functions of the eSTR-containing genes used in the study.

eSTR-Containing Gene	Putative Function	Repeat Motif	Region
comp26672	Protein CHUP1, chloroplastic-like	CCTTCT	coding
comp25013	ATP-dependent Clp protease proteolytic subunit 5, chloroplastic-like	GACGGT	5′UTR
comp41936	ATP synthase delta chain, chloroplastic-like	TCATT	5′UTR
comp45709	cytochrome P450 86A22-like (CYP86A22)	GTGTTT	5′UTR
comp47993	Putative dual specificity protein phosphatase DSP8	TTCAA	5′UTR

## Data Availability

All sequence data from the RNA-Seq experiment have been deposited at the National Center for Biotechnology Information short read archive under project PRJNA408292.

## Supplementary Materials

The following supporting information can be downloaded at https://www.mdpi.com/article/10.3390/biom14080944/s1: Figure S1: Correlation between log transformed relative concentrations of actin and ubiquitin; Figure S2: Correlation between the log transformed relative concentrations of the five eSTR-containing genes and the average concentration of the two control genes (actin and ubiquitin); Table S1: Voucher details for common sunflower (*Helianthus annuus* L.) populations from Nebraska (NE) and Texas (TX) used in this study; Table S2: TaqMan gene expression assays designed for five eSTR-containing genes in *Helianthus annuus* L.; Table S3: CT values of standards used to establish standard curves for the seven assays; Table S4: Standard curve-based estimates for two control and five eSTR-containing genes in *Helianthus annuus*. CT: Cycle threshold; Table S5: Standard curve-based estimates for two control and five eSTR-containing genes in *Helianthus annuus*. CT: Cycle thresholdCT values obtained from the 96 individuals across the seven assays; Table S6: Genotype data from the five eSTR loci used in the study; Table S7: Correlation between relative concentrations of the five eSTR-containing genes and the average concentration of actin and ubiquitin; Table S8: Linear regression models built to assess the effects of eSTR dosage and population on gene expression; Table S9: Results from the ANOVA analysis that estimated the effect size of STR dosage on gene expression; Table S10: Linear regression models built to assess the effect of latitude on normalized eSTR length; Table S11: Results from the ANOVA analysis that estimated the effect size of latitude on normalized STR length; Table S12: Quantiles of gene expression levels by population and locus.
