# Supplementary material for: Clinal Variation in Short Tandem Repeats Linked to Gene Expression in Sunflower (Helianthus annuus L.)
Source: Biomolecules. 2024 Aug 3;14(8):944. doi: 10.3390/biom14080944 (PMC11352406; doi:10.3390/biom14080944)
Supplement: Supplementary file 1 [file biomolecules-14-00944-s001.zip › Supplemental_Figures_biomolecules_R1.pdf]

Clinal variation in short tandem repeats linked to gene expression in sunflower

Chathurani Ranathunge and Mark E. Welch

Supplemental Figures

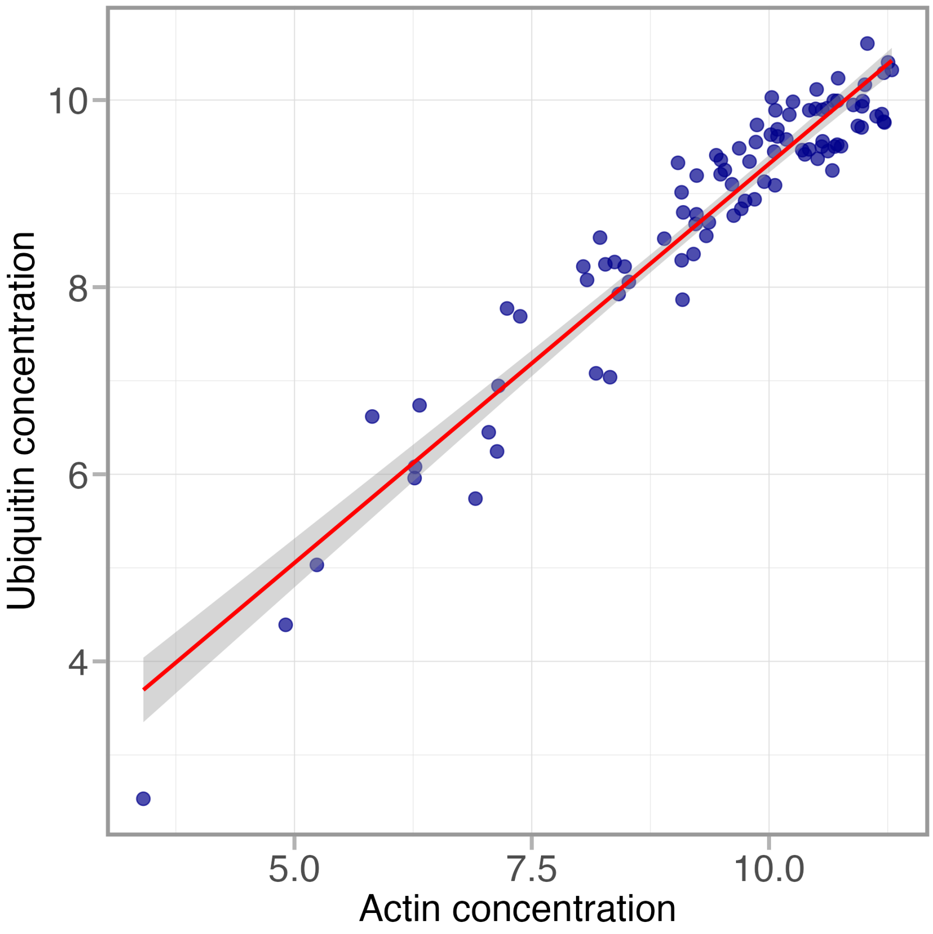

Figure S1: Correlation between log transformed relative concentrations of actin and ubiquitin

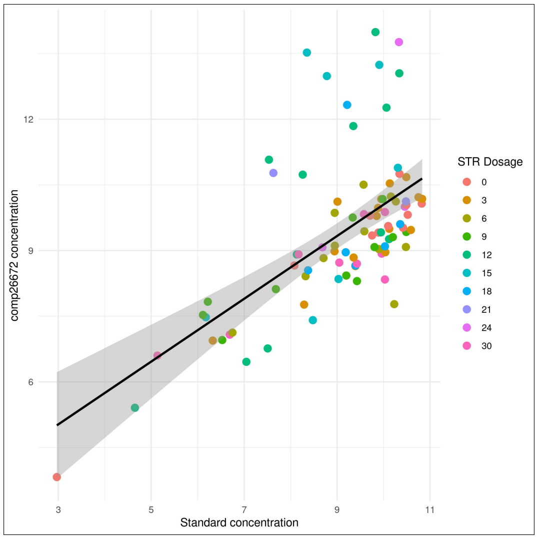

(a) comp26672

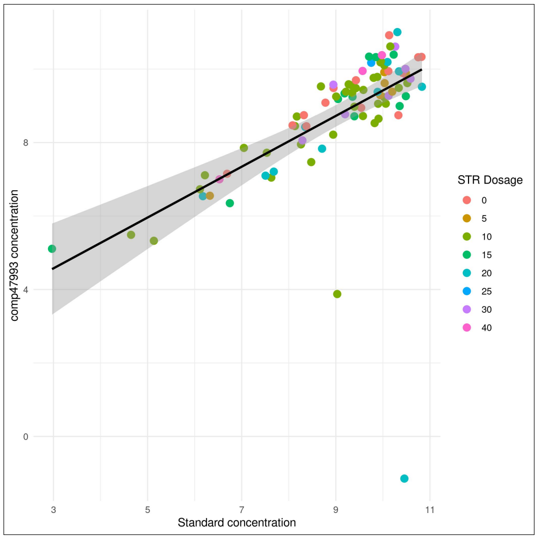

(b) comp47993

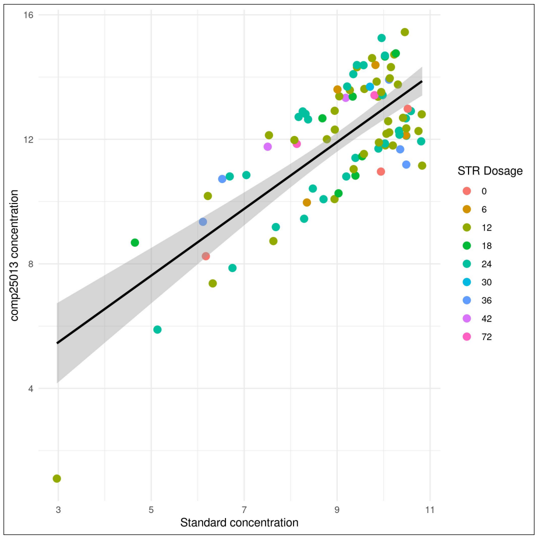

(c) comp25013

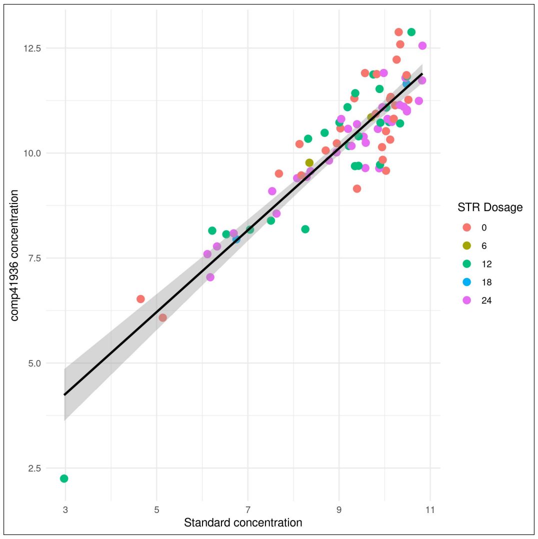

(d) comp41936

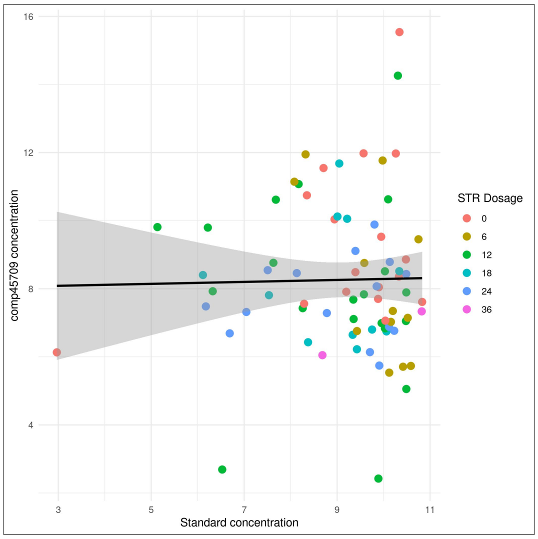

(e) comp45709

Figure S2: Correlation between the log transformed relative concentrations of the five eSTR-containing genes and the average concentration of the two control genes (actin and ubiquitin)
